# Overview of Gas-Generating-Reaction-Based Immunoassays

**DOI:** 10.3390/bios14120580

**Published:** 2024-11-28

**Authors:** Zhao-Jiang Yu, De-Hua Deng, Si-Rui Liang, Ya-Liang Huang, Xin-Yao Yi

**Affiliations:** 1 College of Chemistry and Chemical Engineering, Anyang Normal University, Anyang 455000, China; yzj86jiang@aynu.edu.cn (Z.-J.Y.); lsr052178@163.com (S.-R.L.); 2 School of Pharmacy, Hunan University of Chinese Medicine, Changsha 410208, China; 3 College of Chemistry and Chemical Engineering, Central South University, Changsha 410083, China; yixinyao@csu.edu.cn

**Keywords:** immunoassays, gas generating, pressure, nanozymes, point-of-care testing

## Abstract

Point-of-care (POC) immunoassays have become convincing alternatives to traditional immunosensing methods for the sensitive and real-time detection of targets. Immunoassays based on gas-generating reactions were recently developed and have been used in various fields due to their advantages, such as rapid measurement, direct reading, simple operation, and low cost. Enzymes or nanoparticles modified with antibodies can effectively catalyze gas-generating reactions and convert immunorecognition events into gas pressure signals, which can be easily recorded by multifunctional portable devices. This article summarizes the advances in gas-generating-reaction-based immunoassays, according to different types of signal output systems, including distance-based readout, pressure differential, visualized detection, and thermal measurement. The review mainly focuses on the role of photothermal materials and the working principle of immunoassays. In addition, the challenges and prospects for the future development of gas-generating-reaction-based immunoassays are briefly discussed.

## 1. Introduction

Based on the high specificity and robustness of antigen–antibody interactions, immunoassays have become the gold standard for the selective and sensitive detection of various analytes. They have the advantages of high sensitivity, good universality, and strong on-site monitoring ability for disease diagnosis, environmental protection, and food safety applications [1]. For the ultrasensitive detection of targets, immunoassays have been combined with other powerful signal amplification strategies and detection techniques. According to the type of output signal, immunoassays can be divided into those of fluorescence, colorimetry, chemiluminescence, surface plasmon resonance spectroscopy, surface enhanced Raman spectroscopy, electrochemistry, electrochemiluminescence, photo-electrochemistry, and so on [1,2,3,4,5]. Although their performances are sufficient for the routine analysis of targets, these immunosensing methods still have some inherent limitations, including expensive instruments, highly specialized operators, and/or complex operating procedures [6]. These drawbacks may greatly limit their applications in resource-limited countries or regions. In order to meet the requirements of real-time analysis in resource-constrained environments, point-of-care (POC) testing has been developed rapidly in recent decades [7]. Typically, POC testing systems consist of molecular recognition elements, transducers, and signal processors. At present, the molecular recognition elements include DNA, aptamers, antibodies, peptides, molecularly imprinted polymers, and some specific molecules, such as boric acids and folates [8,9,10]. As an important part of POC testing, biosensors can convert molecular recognition signals into measurement signals, which are processed by signal processors to quantify targets. POC testing has inherent advantages: it is affordable, fast, portable, user-friendly, and deliverable to end-users. Therefore, integrating conventional immunoassays with POC testing devices for personalized medical care, disease diagnosis, and food safety is a new trend.

Immunorecognition events have been transformed into different types of signals using a certain transducer, such as electricity, color, smell, pH, glucose, and temperature, which can be easily observed via the multimeter, the naked eye or nose, a pH meter, a personal glucose meter, or a thermometer. For example, personal glucose meters are normally utilized for glucose detection in human blood samples, but they have been creatively employed to quantify other targets by using glucose as the measured signal molecule [11,12]. Lateral flow strips based on enzymes or nanoparticles, such as commercial pregnancy tests and COVID-19 tests, have been widely developed due to their low cost and user-friendliness [13]. Recently, gas-generating-reaction-based immunoassays have been used in various fields due to their excellent advantages, including rapid measurement, direct signal output, ease of operation, and low cost [14,15]. The combination of immunoreactions with gas-generating reactions can transform the concentration of targets into the amount of gas. The conversion between liquid and gas can result in a large volume expansion and pressure change in a sealed container, greatly amplifying the detection signal.

Several review papers focusing on the combination of gas-generating reactions with bioassays have been published [16,17,18]. For example, Liu et al. summarized achievements in POC testing based on gas-generating reactions in 2018 [16]. Song’s group reviewed the advances of gas-propelled biosensors in which gas pushes an ink bar or propels micromotors [17]. Shi et al. summarized the progress of gas-pressure-based biosensors with enhanced portability and sensitivity [18]. However, no paper has provided a systematic overview of gas-generating-reaction-based immunoassays. Considering the rapid development of immunoassays and the considerable potential of POC testing, this review summarized advances in gas-generating-reaction-based immunoassays. In such methods, immunorecognition is usually combined with a catalyzed gas-generating reaction that can be significantly accelerated by catalysts, such as natural enzymes and nanomaterials. Many types of gas-generating reactions, such as the decomposition of H_2_O_2_, ammonia borane (NH_3_BH_3_), and ammonium bicarbonate (NH_4_HCO_3_), have been successfully used in POC bioassays [19]. A small quantity of gas produced from the specific reaction inside a sealed container will significantly increase the system pressure, which can be easily monitored using different simple and portable devices, such as microfluidic chips, pressure meters, and digital multimeters. In addition, the change in gas pressure can be transformed into other types of signal readout and monitored by electronic balances and thermometers [20,21]. In this review, we summarized the advances of gas-generating-reaction-based immunoassays according to the signal output method, including distance-based readout, pressure differential, visualized detection, and thermal measurement. The targeted scientific resources cover a period from the first report on a gas-generating-reaction-based immunoassay in 2012 until the present. The Web of Science core collection database was employed to collect relevant publications with the keywords “pressure” and “immunoassays” or “immunosensors”. After careful manual selection, relevant works in the field of interest were discussed as examples.

## 2. Types of Gas-Generating Reactions

To date, different types of gas-generating reactions have been used in the design of biosensors. These reactions are generally accelerated by enzymes or nanozymes with high catalytic activities for signal amplification. The speed of gas production has an important influence on the sensitivity and selectivity of biosensors. The gas-generating reactions mainly involve the decomposition of H_2_O_2_, the hydrolysis of NH_3_BH_3_, the catalytic oxidation of luminol, the decomposition of NH_4_HCO_3_, and others. The decomposition of H_2_O_2_ into O_2_ is the most commonly used gas-generating reaction due to its high yield and nontoxic products. Theoretically, 100 μL of H_2_O_2_ (30%, approximately 0.9 mmol of H_2_O_2_) can produce about 0.45 mM of O_2_, which is approximately equivalent to 10 mL of gas. In a 5 mL closed container, it can reinforce the pressure to 200 kPa. However, the rate of spontaneous decomposition of H_2_O_2_ is too slow. Therefore, various materials have been used as catalysts to accelerate the conversion of H_2_O_2_ to O_2_, such as catalase, noble metal nanoparticles, and metal oxides [22,23,24,25].

Although the decomposition of H_2_O_2_ into O_2_ is the most commonly used gas-generating reaction because of its high yield and nontoxic products, the catalytic decomposition reaction is fast and uncontrolled, which may result in gas escaping before the container can be sealed. The hydrolysis of NH_3_BH_3_ is a spontaneous redox reaction occurring under a low reaction temperature. Additionally, NH_3_BH_3_ has a high hydrogen capacity (19.6 wt.%), and the product, H_2_, is nontoxic. Thus, NH_3_BH_3_ has become a promising candidate for hydrogen storage materials and gas-generating-reaction-based biosensors [26]. Theoretically, 1 mmol of NH_3_BH_3_ can produce 67.2 mL of H_2_ gas. When the decomposition reaction occurs in a 1 mL sealed tube, the pressure will increase remarkably, a process that can be easily recorded using a pressure meter. Accordingly, the dehydrogenation of NH_3_BH_3_ can be catalyzed by noble metal nanoparticles and metal oxides [27,28,29]. For example, Wang’s group reported two pressure bioassays for cancer cell detection using CuO/Co_3_O_4_ and CuO-NiO/C heterojunction nanofibers to catalyze the hydrolysis of ammonia borane [28,29]. A CuO/Co_3_O_4_ heterojunction nanofiber was modified with folate to recognize the folate receptor expressed on cancer cells. Under visible-light irradiation, CuO/Co_3_O_4_ could effectively catalyze the hydrolysis of NH_3_BH_3_ to produce H_2_ at room temperature within 4.5 min. Nonetheless, the cost of preparing ammonia borane is relatively high compared to that of H_2_O_2_.

Luminol is one of the most thoroughly investigated luminogenic substrates in chemiluminescence [30]. It is susceptible to oxidation by H_2_O_2_ under the catalysis of horseradish peroxidase (HRP) and nanozymes [31]. The produced intermediate can be further transformed into N_2_ and an excited-state 3-aminophthalate anion. The latter can further return to ground state and emit light for chemiluminescence analysis. Notably, another product, N_2_, is an inert and nontoxic gas that can be used for gas-generating-reaction-based bioassays. However, the N_2_ generation efficiency is lower than in O_2_ generation reactions.

NH_4_HCO_3_ is commonly used as a fertilizer, flavoring agent, or supplier of CO_2_ or NH_3_. It can be decomposed into CO_2_, NH_3_, and H_2_O under temperatures higher than 42 °C. The combination of photothermal materials and the heat-accelerated decomposition of NH_4_HCO_3_ has been used in several gas-generating-reaction-based biosensors [32,33]. For instance, Hu et al. reported a photothermally triggered, controllable, pressure-based aptasensor for cancer cell detection [34]. In this work, hollow, porous, gold nanospheres served as excellent photothermal nanomaterials to produce heat under irradiation with near-infrared light. The increased temperature of the solution resulted in the fast decomposition of NH_4_HCO_3_ into CO_2_ and NH_3_, leading to an increase in pressure. Additionally, urease can catalyze the hydrolysis of urea into NH_3_ and CO_2_, and its activity can be significantly improved at higher temperatures [35,36]. Based on this fact, biosensors have been designed for urea detection using urease-modified, gold nanoflowers [37]. Under illumination with NIR light, gold nanoflowers with a good photothermal effect can generate heat and, thus, improve urease’s activity to catalyze the decomposition of urea into NH_3_ and CO_2_, resulting in enhanced pressure. Despite these advancements, the hydrolysis of NH_4_HCO_3_ and urea is an endothermic process, and the solubility of CO_2_ and NH_3_ is better than that of O_2_ and N_2_. These two factors may lower the detection sensitivity and limit the applications of NH_3_- and CO_2_-generating-reaction-based bioassays.

In addition to the above catalytic gas-generating reactions, the target can react with a probe to directly produce gas within a sealed tube and cause a pressure change. For example, Xiao et al. reported a gas-pressure-based assay for nitrite detection in which nitrite reacted with sodium cyclamate under acidic conditions and produced N_2_ for signal output [38]. However, this type of target-participating reaction is rare, and the generalizability of this method is poor.

## 3. Distance-Based Readout

Immunoassays with distance-based readouts allow for the visual detection of targets, without the requirement of any traditional instruments, such as fluorescence and absorption spectroscopy. The introduction of a gas-generating reaction can translate a molecular recognition event into the production of gas. In a sealed device, the volumetric expansion from the gas-generating reaction can lead to the movement of the ink bar along the channel. The length change will be proportional to the target concentration, which can be measured as simply as reading a thermometer. Nowadays, in combination with microfluidic chips, capillary tubes, and other miniaturized devices, distance-based gas pressure immunoassays have become excellent alternative strategies for POC testing [39,40]. Herein, we summarized the development of distance-based gas pressure immunoassays with microfluidic chips, capillaries, and others (Table 1).

### 3.1. Chip-Based Immunoassays

**Figure 1:**
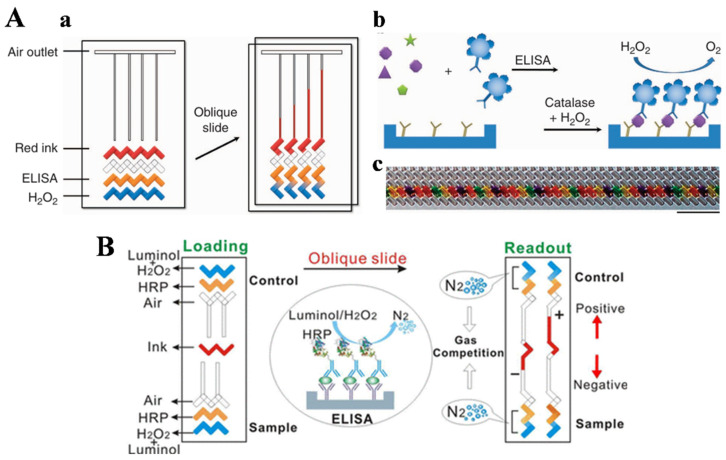
(**A**) Working principle of the V-chip [48]. Copyright 2012 Springer Nature. (**a**) Schematic view of a typical V-chip. On the left is a view of an assembled V-chip with the flow path at the horizontal position. Ink and H_2_O_2_ can be preloaded, and the ELISA assay can be performed in the designated lanes. An oblique slide breaks the flow path and forms the structure on the right, causing catalase and H_2_O_2_ to react and push the inked bars. (**b**) V-chip ELISA reaction scheme and the oxygen generation mechanism. (**c**) Image of 50 sample wells loaded with different-colored food dyes using swab tips. Scale bar, 0.5 cm. (**B**) Schematic illustration of a competitive V-chip with real-time internal control for the detection of hCG based on generation of N_2_ gas [50]. Copyright 2015 American Chemical Society.

Due to their unique advantages of low sample volume, short analysis time, and high sensitivity, microfluidic devices have become ideal platforms for POC testing in past decades [41,42]. As one type of microfluidic chips, volumetric bar-chart chips (V-chip) with ink bar charts to indicate the pressure change resulting from an increased gas volume in channel have elegantly converted molecular recognition events into easily measurable distance readouts for target quantification [43,44,45,46,47]. For instance, Qin’s group first reported a distance-based immunoassay platform for the multiplexed visual detection of protein biomarkers with V-chips (Figure 1A) [48]. In this work, catalase was immobilized on silica nanoparticles (SiO_2_ NPs) to catalyze the decomposition of H_2_O_2_ into H_2_O and O_2_. After the loading of substrate and sample, the slide was titled to facilitate the gas-generating reaction. The produced O_2_ in the limited space of the microfluidic channel resulted in increased gas pressure and propelled the preloaded inked bars into each parallel channel. The length of the ink movement was directly related with the target concentration, allowing for multiplex, visual, and quantitative detection without the use of additional instruments. In addition, Cai et al. developed a microfluidic immunosensor for the visual detection of *S. typhimurium* using immunomagnetic beads and catalase-loaded polystyrene nanospheres [49]. Subsequently, Li et al. constructed a competitive V-chip with real-time internal control for the detection of human chorionic gonadotropin (hCG) based on N_2_ produced from the catalytic decomposition of luminol [50]. As shown in Figure 1B, the conventional ELISA was conducted on the sample and control section in the same channel. Horseradish peroxidase (HRP) conjugated with detection antibody could catalyze the reaction between luminol and H_2_O_2_. The produced N_2_ in direct competition could obviously eliminate possible environmental difference and reduce background influence. However, natural enzymes suffer from several shortcomings, such as the high cost of preparation and purification, low storage and operational stability, and the strict requirement of working conditions. Furthermore, catalase may be destructed during the catalysis of H_2_O_2_ decomposition, and its activity will be reduced in the presence of a high concentration of H_2_O_2_ [51].

**Figure 2:**
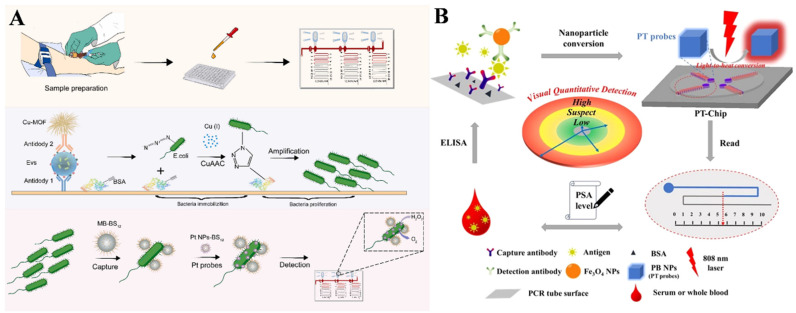
(**A**) Schematic illustration of a V-chip and PtNP-based immunoassay for the visual detection of EVs using a bacteria-proliferation-based cascade amplification strategy [67]. Copyright 2024 Elsevier. (**B**) Schematic illustration of a photothermal bar-chart microfluidic chip for the visual detection of PSA based on nanomaterial-mediated photothermal effects [69].

Since Gao et al. first discovered the intrinsic peroxidase-like activity of ferromagnetic nanoparticles in 2007 [52], various materials have been demonstrated to possess enzyme-like properties, such as organic molecules, noble metal nanomaterials, metal oxides, metal–organic frameworks, and carbon-based nanostructures [53,54,55]. Due to their excellent catalytic capacity for gas production, noble metal nanoparticles, especially platinum nanoparticles (PtNPs) and Pt-based core shells and alloys, have been widely used as gas-generating catalysts in V-chip-based gas pressure assays for the detection of various targets, including heavy metal ions [56], cocaine [57], DNA [58], creatine kinase-MB [59], carcinoembryonic antigen (CEA) [60,61], C-reactive protein (CRP) [62], prostate-specific antigen (PSA) [63], single nucleotide variations [64], and *Escherichia coli* (*E. coli*) [65]. Song et al. developed a gas-generating-reaction-based V-chip immunosensor for the visible and quantitative detection of cancer biomarkers by using PtNPs as the substitutes of catalases [66]. Recently, Li et al. proposed a bacteria-proliferation-based cascade amplification strategy for visual detection of extracellular vesicles (EVs) based on V-chips and PtNPs [67]. As shown in Figure 2A, EVs in blood were captured and labeled through a sandwich immunoaffinity technique, in which the detection-antibody-modified Cu-MOFs were used as signal labels. Upon the addition of ascorbic acid (AA), Cu-MOFs were destroyed and, thus, released a large amount of Cu (I) ions to trigger the click reaction between the azide-labeled bacteria and the acetylene-conjugated bovine serum albumin (BSA) on the substrate. Bacteria immobilized on the substrate were exponentially amplified within a short timeframe. Then, the proliferated bacteria were separated with the aid of magnetic beads (MBs) and further labeled with PtNPs. Finally, the PtNP-catalyzed generation of O_2_ was conducted on the V-chip platform, realizing the visual detection of EVs. However, conventional V-chips exhibit several intrinsic shortcomings, such as costly and complicated procedures for chip preparation and assembly and relatively low controllability of gas production [68]. For this view, Zhou et al. reported a photothermal bar-chart microfluidic chip for the visual detection of PSA by integrating nanomaterial-mediated photothermal effects with V-chips (Figure 2B) [69]. In this study, Fe_3_O_4_ NPs with weak photothermal effect were used as sacrificial labels. After the sandwich immunoassays, Fe_3_O_4_ NPs were converted into Prussian blue (PB) NPs via a simple complexation reaction under an acidic condition. The produced PB NPs possessed the strong absorption in the near-infrared (NIR) region and generated heat under an 808 nm NIR laser. The heat led to a dramatic increase in gas pressure within the microwell and caused the movement of the colored sample solution for visual observation.

### 3.2. Capillary-Based Immunoassays

**Figure 3:**
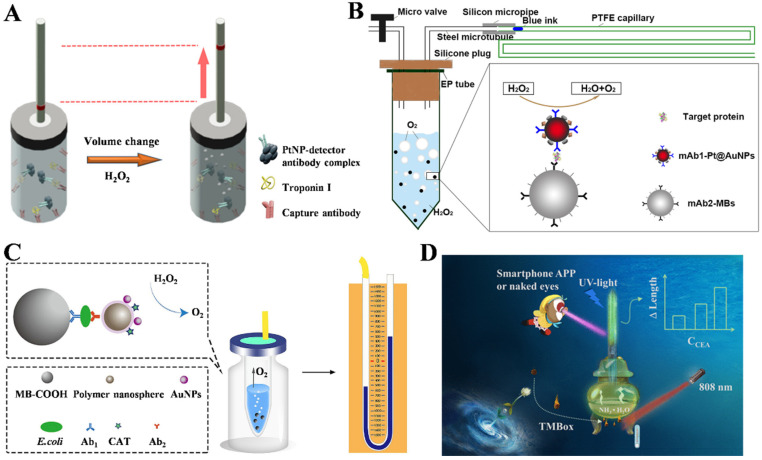
(**A**) Schematic illustration of an POC immunosensor for TnI detection based on dendritic PtNPs and capillary tube indicators [70]. Copyright 2015 American Chemical Society. (**B**) Schematic illustration of a capillary-based immunosensor for PSA detection using Pt@AuNPs as catalytic labels [71]. Copyright 2016 Elsevier. (**C**) Schematic illustration of a pressure immunosensor for the determination of *E. coli* in water using catalase-modified, AuNP-loaded polystyrene nanospheres [72]. Copyright 2020 Elsevier. (**D**) Schematic illustration of a pressure-based immunoassay for CEA detection based on the photothermal effect of oxTMB and the fluorescence quenching of perovskite [73]. Copyright 2022 American Chemical Society.

Besides microfluidic V-chips, simple and cheap capillary tubes can be utilized for the design of gas-generating-reaction-based immunoassays. Typically, Jeon et al. developed a POC immunosensor for the determination of Troponin I (TnI) with dendritic PtNPs nanocatalysts and capillary tube indicators for signal readout (Figure 3A) [70]. In this study, dendritic PtNPs with higher catalytic activity were modified with antibody to capture TnI in human serum and then immobilized on the antibody-modified inner surface of a glass vial. After the addition of H_2_O_2_, the production of O_2_ in a sealed glass vial increased the pressure and improved the ink level in the capillary tube, which was proportional to the concentration of TnI. Similarly, Wu et al. developed a capillary-based immunosensor for PSA determination using Pt@AuNPs as catalytic labels (Figure 3B) [71]. In this study, capture-antibody-modified magnetic beads and detection-antibody-modified Pt@AuNPs were used to specifically capture and label antigens. Pt@AuNPs catalyzed the decomposition of H_2_O_2_ to produce a large volume of O_2_ and drove the movement of glue ink inside a polytetrafluoroethylene capillary. Finally, the distance of ink movement was positively related with the concentration of target proteins (PSA and CEA). To increase the ratio of the antigen-to-enzyme label, Li et al. reported a pressure immunosensor for the determination of *E. coli* in water using catalase-modified, AuNP-loaded polystyrene nanospheres (Figure 3C) [72]. After the immunoreactions, catalase-loaded polystyrene nanospheres were coupled to magnetic immunobeads. Then, many catalase molecules in the immunocomplexes effectively catalyzed the decomposition of H_2_O_2_ to generate a large volume of O_2_. The different height of the liquid between the two tubes in the barometer reflected the level of *E. coli* in the water. However, the methods based on catalase or the PtNP-catalyzed production of O_2_ showed poor controllability. As mentioned above, nanomaterial-mediated photothermal effects can induce a change in gas pressure in a sealed container. A common substrate for colorimetric assays is 3,3′,5,5′-tetramethylbenzidine (TMB), which, along with its oxidized product defined as oxTMB, exhibits excellent photothermal conversion efficiency. Liu et al. proposed a pressure-based immunosensor for CEA assay according to the photothermal effects of oxTMB and the fluorescence quenching of perovskite [73]. As presented in Figure 3D, HRP was used to catalyze the oxidation of TMB by H_2_O_2_. The blue product oxTMB could generate heat under 808 nm laser irradiation, resulting in the increased concentration of ammonia gas. CsPbBr_3_ perovskite was immobilized on the inside wall of the capillary, and its fluorescence was quenched by the released ammonia gas. Finally, the fluorescence quenching length was observed for the visual semiquantitative detection of CEA.

### 3.3. Others

**Figure 4:**
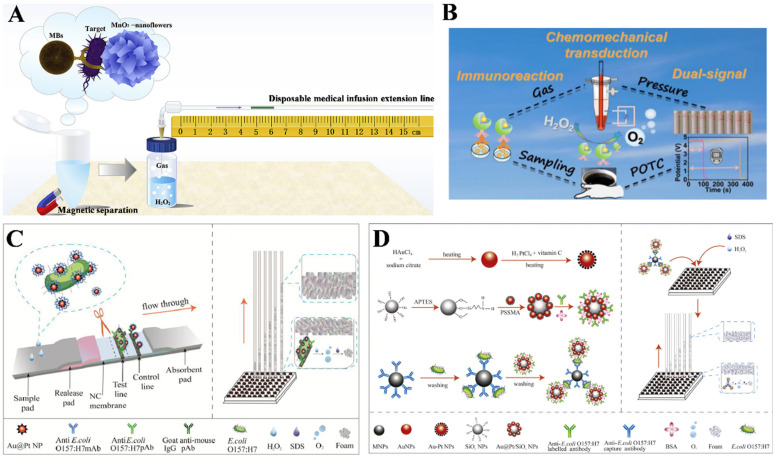
(**A**) Schematic illustration of a POCT immunoassay for the detection of foodborne pathogenic bacteria using MnO_2_ nanoflowers as labels and a disposable medical infusion extension line as readout [76]. Copyright 2019 Elsevier. (**B**) Schematic illustration of a dual-mode pressure immunosensor for the detection of aminopyrine based on color distance change and electrochemical signal [77]. Copyright 2023 American Chemical Society. (**C**) Schematic illustration of a foam-based immunoassay for the detection of *E. coli* O157:H7 using Au@Pt/SiO_2_ NPs [78]. Copyright 2019 Elsevier. (**D**) Schematic illustration of an unplugged foam-based immunochromatographic assay for *E. coli* O157:H7 detection using Au@PtNPs [79]. Copyright 2020 Elsevier.

Metallic oxides and sulfides have diverse properties, including catalytic, magnetic, fluorescence quenching, and dielectric abilities. In recent years, metallic oxides as nanozymes have been utilized in gas-generating-based immunoassays because of their various valence states, natural abundance, and electronic configurations [74]. Deng et al. designed a flexible pressure immunosensor for the determination of silk fibroin by utilizing CuO nanoparticles to catalyze the generation of O_2_ from H_2_O_2_ [75]. Bu et al. reported a POC immunoassay platform for the detection of foodborne pathogenic bacteria using MnO_2_ nanoflowers as labels and a disposable medical infusion extension line for signal readout (Figure 4A) [76]. In this study, MnO_2_ was in situ loaded in organic–inorganic hybrid nanoflowers (MnO_2_ nanoflowers) consisting of Cu_3_(PO_4_)_2_ and concanavalin A (Con A) via a facile coprecipitation method. After the target bacteria in samples was captured and separated by antibody-modified magnetic beads, MnO_2_ nanoflowers were used to selectively label bacteria via the specific recognition between Con A and O-antigen on the bacterial surface. Then, the solution was transferred into a sealed glass vial. MnO_2_ nanoflowers with catalase-mimicking activity catalyzed the decomposition of H_2_O_2_ to O_2_, and the increased gas pressure led to a movement of dye solution in the disposable infusion extension line that was connected to the glass vial. The distance that solution moved in the line was proportional to the bacteria concentration. The detection limit for the detection of both *E. coli* O157:H7 and *Salmonella* sp. was 10 CFU/mL. In addition, Chen et al. fabricated a dual-mode pressure immunosensor for the assay of aminopyrine, according to the changes in color distance and electrochemical signal (Figure 4B) [77]. In this study, core-shell CeO_2_@Pt/Au showing improved catalytic activity was modified with antibodies. In the presence of aminopyrine, the competitive immunoassay was conducted in the 96-well plate. After that, the unconjugated solution was transferred into a sealed reaction vessel to further trigger the generation of O_2_ from H_2_O_2_. The increased pressure in a sealed vessel caused the movement of the red oil phase solution from the bottom to the coil. The change from the oil phase to the water phase around the electrode resulted in the sudden change in potential.

During the “Elephant’s Toothpaste” experiment, O_2_ produced from the rapid decomposition of H_2_O_2_ in the presence of surfactant can produce a great amount of foam. Thus, the gas-induced formation of foam can be combined with the catalytic generation of gas in pressure immunoassays. The foam height will be positively related to the target concentration, which can be readily determined by a ruler. For instance, Liu et al. reported a foam-based immunoassay for the determination of *E. coli* O157:H7 (Figure 4C) [78]. In this work, Au@Pt NPs were used as nanocatalysts to promote the decomposition of H_2_O_2_ into O_2_, which could produce 20-fold as much O_2_ per second as catalase. To further amplify the detection signal and improve the stability, Au@Pt NPs were immobilized on silica nanoparticles (SiO_2_ NPs, Au@Pt/SiO_2_ NPs). The sandwich immunoassay was conducted in the presence of capture-antibody-labeled MBs and detection-antibody-labeled Au@Pt/SiO_2_ NPs. In the presence of surfactant sodium dodecyl sulfate, O_2_ generated from the Au@Pt NP-catalyzed decomposition of H_2_O_2_ produced a great amount of foam to propel the foam into the acrylic tube. The foam height change was used to indicate the amount of *E. coli* O157:H7 with a ruler. Furthermore, to simplify the operation procedure and to save time, they integrated the foam-based immunoassay with immunochromatographic trip for *E. coli* O157:H7 detection (Figure 4D) [79]. HRP was used to replace BSA to modify Au@PtNPs. The resulting Au@PtNPs synergized with HRP promoted the decomposition of H_2_O_2_ into O_2_ more efficiently. The generated O_2_ could produce a large amount of foam in the presence of sodium dodecyl sulfate. The height of the foam column in the acrylic tube was measured by a ruler for the quantitative detection of *E. coli* O157:H7.

**Table 1:** Overview of gas-generating-reaction-based immunoassays through distance-based readout.

Signal Label	Target	Linear Range	Detection Limit	Ref.
PtNPs	cTnI, CK-MB, Myo	0–25 ng/mL, 0–33 ng/mL, 0–250 ng/mL	0.014 ng/mL, 0.16 ng/mL, 0.85 ng/mL	[14]
PtNPs/SiO_2_	AFP	0.05−132 ng/mL	16 pg/mL	[15]
Catalase	CRP	200−2000 ng/mL	200 ng/mL	[19]
PtNPs	CEA	7.81−500 pg/mL	0.6 pg/mL	[21]
PtNFs	*E. coli O157:H7*, *S. typhimurium*	10^2^–10^4^ CFU/mL, 10–10^5^ CFU/mL	15 CFU/mL, 7 CFU/mL	[39]
Au@PtNPs	CRP	0.05–6.25 ng/mL	41 pg/mL	[40]
Catalase/SiNPs	*S. typhimurium*	10–10^4^ CFU	10 CFU	[43]
Catalase	*S. typhimurium*	5 × 10^2^–5 × 10^4^ CFU	1.6 CFU	[44]
Catalase	*S. typhimurium*	10^2^–10^6^ CFU/mL	150 CFU/mL	[49]
HRP	hCG	Not reported	1.4 ng/mL	[50]
PtNPs	TnI	0.1–100 ng/mL	0.1 ng/mL	[59]
PtNPs	BNP	4.6–404.7 pM	6 pM	[60]
PtNPs	CEA	Not reported	10 pg/mL	[61]
PtNPs	CRP	25–100 ng/mL	25 ng/mL	[62]
PtNPs	PSA	0–12 ng/mL	0.54 ng/mL	[63]
PtNPs	CYFRA 21-1	0.5–50 ng/mL	0.5 ng/mL	[66]
PB NPs	PSA	2−64 ng/mL	2 ng/mL	[68]
Fe_3_O_4_/PB NPs	PSA	0−64 ng/mL	2.1 ng/mL	[69]
Pt@AuNPs	PSA, CEA	0.02−2.5 ng/mL, 0.063−16 ng/mL	17 pg/mL, 44 pg/mL	[71]
Catalase@AuNPs	*E. coli* *O157:H7*	10^2^–10^7^ CFU/mL	80 cfu/mL	[72]
HRP/TMB	CEA	0−20 ng/mL	78 pg/mL	[73]
CuO NPs	Silk fibroin	10−10^5^ ng/mL	10.58 ng/mL	[75]
MnO_2_ NFs	*E. coli O157:H7*	10–10^5^ CFU/mL	10 CFU/mL	[76]
CeO_2_@Pt/AuNPs	Aminopyrine	0.001−10 ng/mL	0.41 pg/mL	[77]
Au@PtNPs	*E. coli O157:H7*	1.19 × 10^3^–1.19 × 10^7^ CFU/mL	216 CFU/mL	[78]

Abbreviation: PtNPs, platinum nanoparticles; cTnI, cardiac troponin I; CK-MB, creatine kinase-MB isoform; Myo, myoglobin; AFP, alpha fetoprotein; CRP, C-reactive protein; CEA, carcinoembryonic antigen; PtNFs, Pt-nanoflowers; Au@PtNPs, core-shell Au@Pt nanoparticles; *E. coli* O157:H7, *Escherichia coli O157:H7*; *S. typhimurium*, *Salmonella typhimurium*; HRP, horseradish peroxidase; hCG, human chorionic gonadotropin; BNP, B-type natriuretic peptide; PSA, prostate-specific antigen; CYFRA 21-1, cytokeratin 19 fragment; PB NPs, Prussian blue nanoparticles; AuNPs, gold nanoparticles; TMB, 3,3′,5,5′-tetramethylbenzidine.

## 4. Pressure Differential

A pressure meter, a convenient, chip, and portable device, holds great potential in POC assays. In a sealed container, the production of gas will increase the pressure, which can be easily measured with a pressure meter. Thus, bioassays using a pressure meter for signal readout have been reported for the detection of different targets because of their wide detection range and high accuracy, such as spermine [80], alcohol [81], microRNAs [82,83], thrombin [84], cells [34,85], and *E. coli* O157:H7 [86]. For example, Wang’s group reported two pressure bioassays for cancer cell detection using CuO/Co_3_O_4_ and CuO-NiO/C heterojunction nanofibers to catalyze the hydrolysis of ammonia borane [28,29].

**Figure 5:**
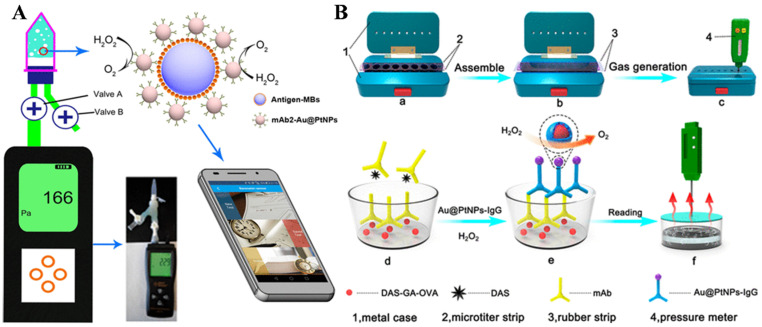
(**A**) Schematic illustration of a Au@Pt core/shell NP-based immunosensor for the detection of CEA and ractopamine with a barometer as readout [96]. Copyright 2017 American Chemical Society. (**B**) Schematic illustration of a competitive-type pressure immunosensor for DAS detection based on Au@PtNPs [97]. Copyright 2019 American Chemical Society.

The combination of enzymes or nanozyme-based, gas-generating reactions and pressure meters has expanded the pressure-based immunoassays of myoglobin (Myo) [87,88,89], HER-2 extracellular domain [90], and *E. coli* O157: H7 [91], as shown in Table 2. For example, PtNPs were in situ embedded in peptide–Cu_3_(PO_4_)_2_ hybrid nanocomposites and used as signal labels to catalyze the hydrolysis of H_2_O_2_ [91]. In 2015, Yang and co-workers first reported a gas-pressure-based immunosensor for PSA detection [92], in which catalase in the sandwich immunocomplex catalyzed the breakdown of H_2_O_2_ to generate O_2_. This led to a significant pressure increase in the sealed vessel, which could be measured by a simple and low-cost pressure meter. Then, they developed a catalase-based immunosensor for the detection of CRP using a portable pressure meter [93]. Based on the similar working principle, Pt-based nanozymes have been used as catalase-like mimics in pressure immunoassays [94]. Ji et al. developed a pressure-based immunosensor for CRP determination utilizing PtNPs to break H_2_O_2_ into O_2_, in which the pressure was related to CPR concentration and then measured by a pen-like pressure meter [95]. Fu et al. reported barometer-based immunosensors for the assays of CEA and ractopamine based on Au@Pt core/shell NPs [96]. As shown in Figure 5A, antibody-modified MBs and Au@PtNPs were used for sandwich immunoassays in plastic centrifuge tubes. After the immunoreaction, Au@PtNPs catalyzed the generation of O_2_, and the increased pressure was measured by a barometer. Aiming to facilitate the POC testing, smartphone software was used to calculate and transmit the results. Tang et al. reported a competitive-type pressure immunosensor for the sensitive determination of diacetoxyscirpenol (DAS) in wheat (Figure 5B) [97]. DAS was labeled with ovalbumin (OVA) using hemiglutarate (HG) as the linker. The formed DAS-HG-OVA was used as an artificial antigen. After the competitive immunoreaction in the presence of DAS, goat anti-mouse antibody (IgG)-functionalized Au@PtNPs were used to label monoclonal antibodies on the plate. Au@PtNPs effectively catalyzed the decomposition of H_2_O_2_ to O_2_, and the increased pressure in the sealed micropore was detected by a pressure gauge. However, the abovementioned pressure-meter-based methods may suffer from time-consuming washing and incubation steps, limiting their applications in POC immunoassays.

**Figure 6:**
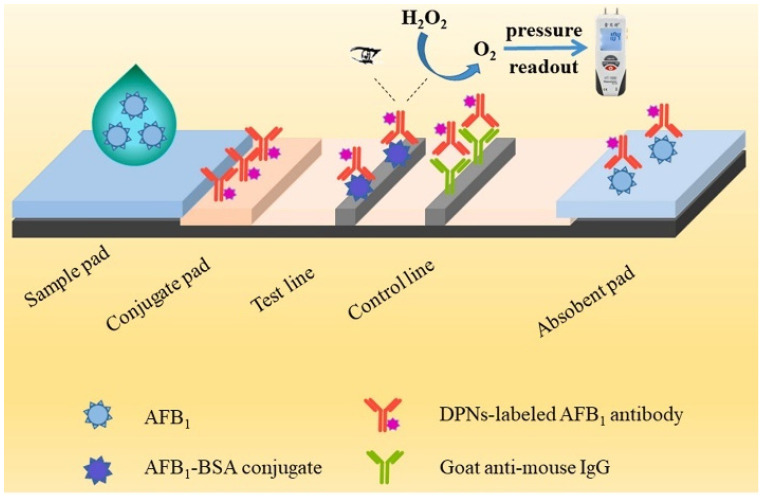
Schematic illustration of a pressure/colorimetric dual-readout ITS for the detection of AFB_1_ [100]. Copyright 2021 Elsevier.

Recently, an immunochromatographic test strip (ITS) has been widely used in different fields, including disease diagnosis, food safety, and environmental monitoring, due to its excellent advantages of low cost, high portability, and ease of operation [98]. Moreover, the combination of pressure-based detection with visual detection can significantly improve the sensitivity and expand its practical applications [99]. For instance, Jiang et al. reported a pressure/colorimetric dual-readout ITS device for the determination of aflatoxin B1 (AFB_1_) [100]. As shown in Figure 6, antibody-modified dendritic PtNPs were used as the signal tracers of immunochromatographic process. After the competitive immunoreaction, PtNPs immobilized on the test line produced a black color, allowing for the colorimetric qualification of AFB_1_. The test line was cut and transferred into a sealed tub. Dendritic PtNPs effectively catalyzed the decomposition of H_2_O_2_ and led to an obvious pressure change, which could be monitored by a hand-held pressure meter.

**Figure 7:**
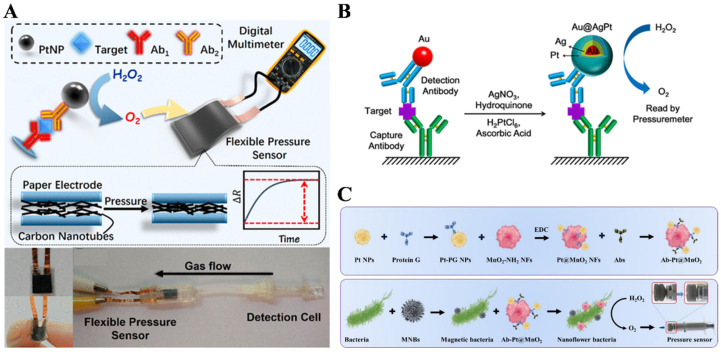
(**A**) Schematic illustration of a PtNP-based POC immunoassay based on paper electrode-based flexible pressure sensor and digital multimeter [107]. Copyright 2019 American Chemical Society. (**B**) Schematic illustration of a AuNP-labeled pressure immunoassay using a Pt staining method to stain AuNPs with a Pt shell [109]. Copyright 2018 American Chemical Society. (**C**) Schematic illustration of a pressure immunoassay for *Salmonella* detection using PtNP-loaded MnO_2_ nanoflowers and a thin-film pressure detector [112]. Copyright 2020 Elsevier.

The flexible pressure sensors can precisely convert pressure into an electrical signal, such as current or resistance, which can be monitored by digital multimeters [101]. The sensors have attracted intensive attention during the past few years due to their high sensitivity, extended durability, simpler fabrication processes, and fast response. Recently, Tang’s group reported a series of pressure immunoassays based on PtNPs and flexible pressure sensors for the detection of CEA and prostate-specific antigen (PSA) [102,103,104,105,106]. Additionally, Yu et al. reported a PtNP-based POC immunoassay platform with a paper-electrode-based flexible pressure sensor and digital multimeter, and the protocol for the proposed method is shown in Figure 7A [107]. The secondary antibody-modified PtNPs attached on the microplate efficiently catalyzed the decomposition of H_2_O_2_ to constantly produce O_2_. A large amount of O_2_ flowed into the homemade pressure-tight system and reinforced the pressure of the multiwalled CNT-functionalized paper electrode, which was easily recorded by a digital multimeter. PtNPs are confronted with nonspecific adsorption on porous surfaces, thus leading to a high background signal. However, PtNPs are highly prone to aggregate and easily poisoned during long-term storage, inhibiting their catalytic activities. It has been reported that Au can enhance the catalytic activity of Pt with synergistic and electronic effects [108]. For this consideration, a AuNP-labeled immunoassay was integrated with a Pt staining method to stain AuNPs outside Pt shells [109]. As shown in Figure 7B, the detection-antibody-labeled AuNPs were used for sandwich immunoassays in a microtiter plate, and the nonspecific adsorption on AuNPs was blocked by the blocker of bovine serum albumin. Then, AuNPs were sequentially stained with Ag and Pt bimetallic shells (Au@AgPtNPs) in the presence of a metal precursor and a reductant. The freshly formed Au@AgPtNPs catalyzed the decomposition of H_2_O_2_ to generate O_2_, and the increased pressure inside the sealed microtiter well was recorded by a portable pressure meter. In addition, the Pt staining method was introduced into traditional AuNP-based test strips for the detection of Myo with a simple and portable pressure meter for signal readout [110].

MnO_2_ nanoflowers with a high specific surface area can serve as excellent nanocarriers to load other functional materials [111]. Wang et al. proposed a pressure immunoassay for *Salmonella* detection utilizing PtNP-loaded MnO_2_ nanoflowers and a thin-film pressure detector [112]. As shown in Figure 7C, PtNPs modified with Protein G were immobilized on MnO_2_ nanoflowers (Pt@MnO_2_ NFs), allowing for the oriented immobilization of detection antibodies (Dab). The capture-antibody-modified magnetic nanobeads were employed to selectively capture *Salmonella* and DAb-Pt@MnO_2_ NFs. The Pt@MnO_2_ NFs in the immunocomplexes could catalyze the decomposition of H_2_O_2_ into O_2_ in a sealed centrifuge tube. The increased pressure was recorded with a thin-film piezoresistor using a pressure detector, and the date was proceeded with a smartphone for the quantitative detection of *Salmonella*.

**Figure 8:**
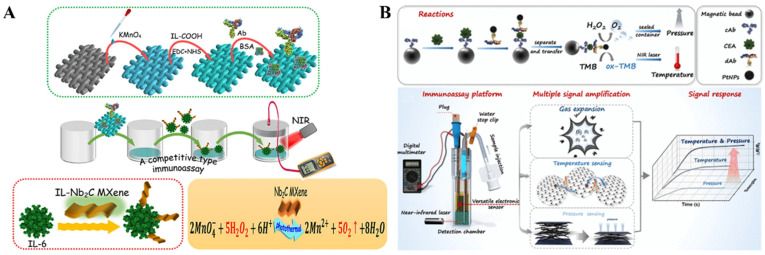
(**A**) Schematic illustration of a multifunctional Nb_2_C MXene-based photothermal-enhanced pressure immunoassay for the portable detection of IL-6 [116]. Copyright 2023 Elsevier. (**B**) Schematic illustration of an enhanced pressure-based immunoassay based on the PtNP-catalyzed chromogenic reaction between H_2_O_2_ and TMB [117]. Copyright 2021 American Chemical Society.

The combination of the photothermal effect with a pressure-based assay has been considered as a promising strategy for amplifying the pressure signal [37,106,113,114]. Recently, several nanozymes with peroxidase-like properties have been reported to possess excellent photothermal effects for photothermal and pressure bioassays, such as polydopamine-functionalized AuPtNPs@CuS nanosheets [33] and chitosan-modified silicon carbide nanoparticles [115]. For instance, Gao et al. suggested a photothermal-enhanced pressure immunoassay for the portable determination of interleukin-6 (IL-6) based on multifunctional Nb_2_C MXene (Figure 8A) [116]. Nb_2_C MXene with good biocompatibility and rich functional groups on its surface was modified with IL-6 antigen. After the competitive immunoreaction on KMnO_4_-modified filter paper, Nb_2_C MXene with excellent peroxidase-like activity could accelerate the generation of O_2_ from H_2_O_2_. Nb_2_C MXene with photothermal effects could increase the temperature in the sealed device under the irradiation of an 808 nm NIR laser and significantly promote the generation of O_2_, finally boosting the pressure signal. Besides, Huang et al. reported a pressure-based immunoassay platform based on PtNP-catalyzed chromogenic reactions between H_2_O_2_ and TMB [117]. As shown in Figure 8B, the capture-antibody-modified MBs were used to capture CEA and detection-antibody-labeled PtNPs by the formation of sandwich immunocomplexes. After magnetic separation and washing, the sandwich immunocomplexes were transferred into a sealed chamber. PtNPs catalyzed the oxidation of TMB by H_2_O_2_ and produced a large amount of O_2_ and the photothermal reagent oxTMB. Under the irradiation of an 808 nm NIR laser, the resulting oxTMB induced an increase in the temperature and, meanwhile, significantly boosted the pressure in the closed device. The increase in pressure and temperature obviously reduced the resistance of a piezoresistive pressure sensor.

**Table 2:** Overview of gas-generating-reaction-based immunoassays with a pressure meter for signal readout.

Signal Label	Target	Linear Range	Detection Limit	Ref.
PtNPs	Myo	2.9–75 ng/mL	2.9 ng/mL	[87]
PtNPs	Myo	0–100 ng/mL	0.26 ng/mL	[88]
PtNPs	sHER-2 ECD	2–50 ng/mL	2 ng/mL	[90]
PtNPs@Cu_3_(PO_4_)_2_	*E. coli* O157:H7	10–10^4^ CFU/mL	10 CFU/mL	[91]
Catalase	CRP	10–50 nM	1.8 nM	[93]
Pt/SiO_2_ NSs	AFP	10–200 ng/mL	3.4 ng/mL	[94]
PtNPs	CRP	5–400 ng/mL	1.6 ng/mL	[95]
Au@PtNPs	CEA	0.025−1.6 ng/mL	21 pg/mL	[96]
PdCuPt NPs	ICP, CAB	0.02−50 ng/mL, 0.15−50 ng/mL	6.7 and 30.2 pg/mL	[99]
DPNs	AFB1	0.05–10 ng mL	30 pg/mL	[100]
PtNPs	CEA	0.2–1000 ng/mL	0.14 ng/mL	[102]
PtNPs	CEA	0.1−40 ng/mL	87 pg/mL	[103]
PtNPs	CEA	0.2–60 ng/mL	0.13 ng/mL	[104]
PtNPs	PSA	0.02–50 ng/mL	12.6 pg/mL	[105]
Catalase	PSA	0.1–50 ng/mL	57 pg/mL	[106]
PtNPs	CEA	0.5−60 ng/mL	167 pg/mL	[107]
Au@AgPtNPs	H5N1	0–3 ng/mL	15 and 65 pg/mL	[109]
Pt@MnO_2_ NFs	Salmonella	1.5 × 10–1.5 × 10^5^ CFU/mL	13 CFU/m	[112]
PtNPs	Myo	6.67–150 ng/mL	3.82 ng/mL	[110]
SiC-CS@Ag	E6 protein	10^−6^–1 ng/mL	1.6 fg/mL	[115]
Nb_2_C MXene	IL-6	10^−5^ to 1 ng/mL	3.33 × 10^−6^ ng/mL	[116]
PtNPs	CEA	0.1–90 ng/mL	81 pg/mL	[117]

Abbreviation: PtNPs, platinum nanoparticles; Myo, myoglobin; sHER-2 ECD, HER-2 extracellular domain; *E. coli* O157:H7, *Escherichia coli O157:H7*; CRP, C-reactive protein; NSs, nanospheres; AFP, alpha fetoprotein; CEA, carcinoembryonic antigen; ICP, imidacloprid; CAB, carbendazim; PSA, prostate-specific antigen; DPNs, dendritic platinum nanoparticles; AFB_1_, aflatoxin B1; H5N1, avian influenza hemagglutinin 5 neuraminidase 1; SiC-CS, chitosan-decorated silicon carbide nanoparticles; IL-6, interleukin-6.

## 5. Visualized Detection

**Figure 9:**
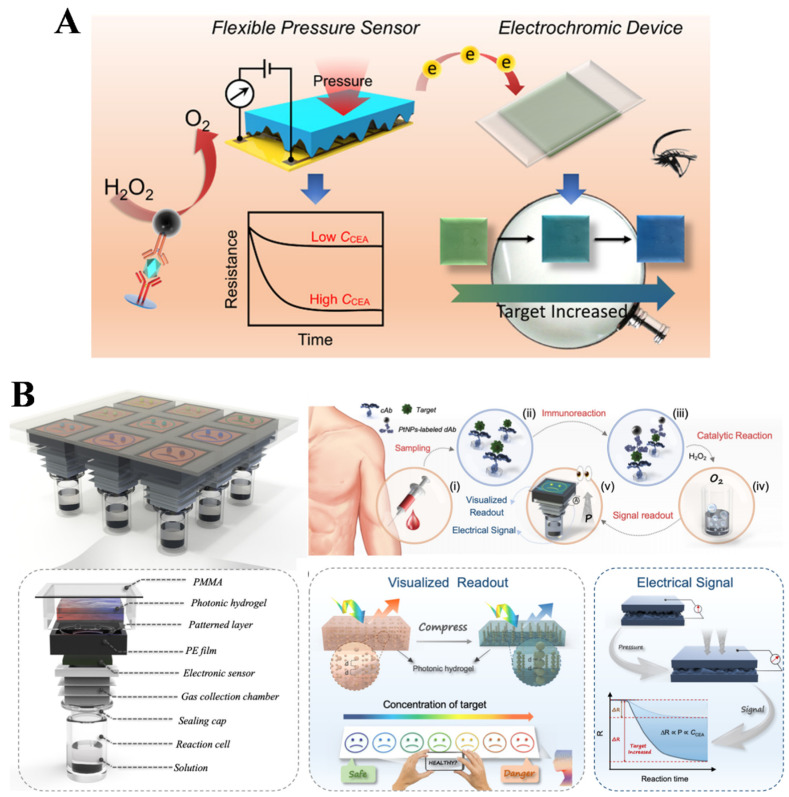
(**A**) Schematic illustration of a PtNP-based pressure immunosensor for the visual detection of CEA combining with a flexible pressure sensor and an electrochromic device [118]. Copyright 2021 American Chemical Society. (**B**) Schematic illustration of a multipixel dual-channel pressure sensor array for CEA detection with the visual sensing ability of full-color switching and electrical signals [119]. Copyright 2022 American Chemical Society.

Methods based on a single-signal detection mode may lack accuracy, reliability, and repeatability due to the interference from different instruments and nonstandard analytical processes. Color, as a visual perception for visual detection, can be directly observed by the naked eye without the use of any equipment. Thus, the integration of pressure immunoassay with visual detection exhibits great potential in POC testing (Table 3). For example, Yu et al. constructed a PtNP-based pressure immunosensor for the visual detection of CEA by combining a flexible pressure sensor with an electrochromic device [118]. As illustrated in Figure 9A, a sandwich-type immunoreaction was conducted in a reaction cell, in which the capture-antibody-modified PtNPs were used as functional labels. PtNPs catalyzed the decomposition of H_2_O_2_ to produce O_2_, and the pressure in the sealed chamber significantly increased, which was monitored by a skin-inspired flexible pressure sensor in real time. Meanwhile, the platform was coupled to a voltage-regulated electrochromic device that was composed of polyaniline and tungsten oxide. The voltage generated from the pressure sensor caused a change in the color of the electrochromic device from green to blue, achieving the visual detection of CEA. Nonetheless, the method required the use of a power supply and complicated wiring connection, and the application of the one-sample-at-a-time detection method was limited in the determination of multiple samples. To address these problems, Huang et al. developed a multipixel dual-channel pressure sensor array with the visual sensing ability of full-color switching and electrical signals [119]. As shown in Figure 9B, the dual-channel pressure sensor was assembled with photonic hydrogels based on chromatic transition and piezoresistive pressure sensor as the electrical data transmission units. Both units had the capability of pressure-induced mechanical stimulus feedback. Target CEA in blood samples was determined in the sandwich-type immunoassay with detection-antibody-modified PtNPs as labels to catalyze the production of O_2_ from H_2_O_2_. The produced gas resulted in an obvious pressure increase in a closed chamber and induced a spatiotemporal change. The dual-channel pressure sensor converted the spatiotemporal stimuli into an accurate electrical signal output and an eye-readable coloration, realizing the dual-signal detection of CEA in blood samples.

## 6. Thermal Measurement

**Figure 10:**
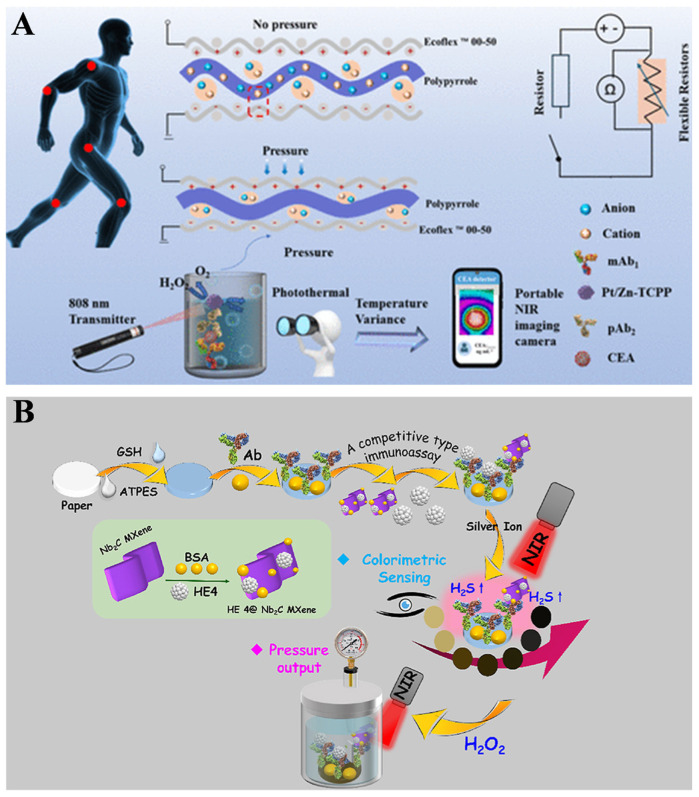
(**A**) Schematic illustration of a flexible sensing platform using Pt/Zn-TCPP nanozyme for dual-mode pressure and the temperature detection of CEA [120]. Copyright 2024 American Chemical Society. (**B**) Schematic illustration of a pressure-colorimetric multisignal immunoassay based on photothermal-activated multiple rolling signal amplification for HE4 detection [122]. Copyright 2023 Elsevier.

A thermometer, a commonly used device in daily life, is usually used as a signal readout of immunoassays. When photothermal nanomaterials are introduced into the sensing system, the light energy is converted into heat energy, accelerating the generation of gas and enhancing the pressure of a sealed system. Thus, the thermal change can be combined with a pressure signal for the design of photothermal and pressure immunoassays with improved accuracy. MOFs, a large family of crystalline porous materials, consist of metal ions/nodes and organic linkers. Recently, it has been reported that MOFs show versatile enzyme-like catalytic abilities, such as peroxidase, oxidase, superoxide dismutase, and hydrolase [124,125]. Meanwhile, owing to their high porosity and large surface area, MOFs have been considered as one of the most promising materials to effectively load other nanozymes for multiplex signal amplification [126]. To avoid the experimental errors and nonstandard analytical procedures in single-mode detection, Wu et al. constructed a flexible sensing platform using PtNPs/zinc-meso-tetrakis(4-carboxyphenyl)-porphyrin (Pt/Zn-TCPP) nanozymes for the pressure and temperature dual-mode detection of CEA (Figure 10A) [120]. In this work, PtNPs were in situ growth on the surface of Zn-TCPP and subsequently coated with polyclonal antibodies. The formed Pt/Zn-TCPP nanozymes exhibited enhanced peroxidase-like catalytic activity due to the synergistic effect, and Zn-TCPP showed excellent photothermal conversion efficiency. In the presence of CEA, the polyclonal antibody-modified Pt/ZnTCPP nanozymes were captured by the monoclonal antibody-modified microplate. Pt/ZnTCPP catalyzed the decomposition of H_2_O_2_ into O_2_ and increased the pressure that was recorded by a polypyrrole-film-based flexible sensor. Meanwhile, Pt/Zn-TCPP caused the increase in temperature under 808 nm NIR irradiation, and the temperature change was monitored easily by a portable NIR imaging camera on a smartphone.

The in-situ generation of photothermal materials is a promising strategy to develop novel pressure and photothermal immunoassays. Typically, Song et al. suggested photothermal and pressure-based immunoassays for the detection of CRP through the in-situ generation of polypyrrole [121]. In this study, the detection antibody-modified magnetic beads were implemented into conventional sandwich-type immunoassays. After treatment with HCl solution, Fe^3+^ ions released from magnetic beads catalyzed the polymerization of pyrrole into polypyrrole that could convert NIR light into heat and further cause the pressure increase. The changes in temperature and pressure can be collected by digital thermometer and pressure meter, respectively. In addition, Chen et al. reported a pressure-colorimetric multisignal immunoassay via photothermal-activated multiple rolling signal amplification for determining human epididymis protein 4 (HE4) [122]. The working principle is depicted in Figure 10B. The filter paper was decorated with GSH and further modified with HE4 antibody. After the competitive immunoreaction, HE4 antigen-modified Nb_2_C MXene was immobilized on the antibody-modified paper. Then, the solution of Ag^+^ ions was dropped on the immunocomplex-anchored paper to generate Ag-GSH complexes. Upon NIR laser radiation, Nb_2_C MXene with photothermal effect could rapidly increase the temperature and cause the decomposition of Ag-GSH complex to form Ag-S_x_ on the surface of Nb_2_C MXene. The formed Nb_2_C MXene/Ag-Sx hybrid showed an enhanced photothermal effect, and the color of the nanocomposite changed from pale yellow to dark brown. Next, the substrate was sealed into the pressure device, followed by treatment with H_2_O_2_. The photothermal effect was enhanced, and the catalase-like activity was activated by rolling in Nb_2_C MXene/Ag-Sx hybrid under 808 nm laser radiation. This improved the rate of H_2_O_2_ decomposition and elevated the pressure inside the sealed device.

**Figure 11:**
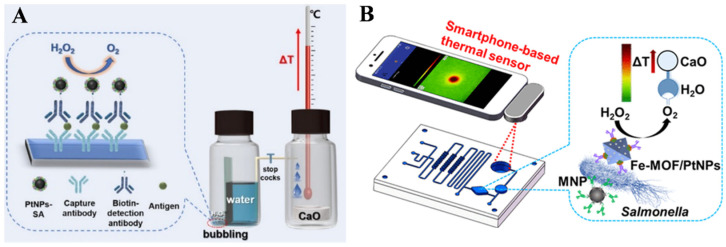
(**A**) Schematic illustration of a thermal immunosensor using a simple thermometer as readout by combing the gas-generating reaction with the exothermic reaction between H_2_O and CaO [21]. Copyright 2019 American Chemical Society. (**B**) Schematic illustration of an immunoassay for *Salmonella* detection based on PtNP-loaded Fe-MOFs as catalysts and the combination of the gas-generating reaction with the exothermic reaction [123]. Copyright 2021 American Chemical Society.

Target-triggered pressure change can be converted into temperature change by a certain exothermic reaction [127]. Ma et al. reported a thermal immunosensor employing a simple thermometer as the signal recorder by integrating the gas-generating reaction with the exothermic reaction between H_2_O and CaO [21]. As presented in Figure 11A, an immunosorbent assay was conducted on the target recognition area and the sandwich immunocomplex formed in the presence of target CEA. Streptavidin (SA)-modified PtNPs were used to label the biotin-conjugated antibody through biotin–SA interaction. In the presence of H_2_O_2_, the pressure in the sealed vial was elevated due to the PtNP-catalyzed generation of O_2_. When the stopcock was switched on, a certain amount of water flowed into the exothermic reaction bottle because of the pressure differential. The exothermic reaction between H_2_O and CaO produced a large amount of heat, and the temperature change was monitored by a common thermometer. Additionally, Guo and co-workers reported an immunoassay strategy for *Salmonella* detection utilizing PtNP-loaded Fe-MOFs (Fe-MIL-88NH_2_) as catalysts with a combination of the gas-generating reaction and the exothermic reaction [123]. As presented in Figure 11B, Fe-MIL-88NH_2_ was used to load PtNPs (Fe-MOF/PtNPs) and further modified with detection antibodies via click reaction. Target *Salmonella* was specifically grasped by capture-antibody-modified magnetic nanoparticles (MNPs) and then labeled with detection-antibody-modified Fe-MOF/PtNPs. After the sandwich complexes were separated and washed, H_2_O_2_ solution was injected into the chamber, producing a large amount of O_2_ and pushing the water into the heating chamber. The exothermic reaction between CaO and H_2_O caused the release of heat, which was measured by the smartphone-based thermal sensor.

## 7. Conclusions and Perspectives

This article provides an overview of gas-generating-reaction-based immunoassays in recent years. Innovatively designing various types of gas signal readout systems is critical for the development of such immunoassays. In particular, we focus on the strategies of combining natural enzyme- and nanozyme-based, gas-generating reactions with various signal readout devices, which greatly increases the variety of gas-generation-based immunoassays. Compared with conventional immunoassays, gas-generating-reaction-based immunoassays have several advantages. For example, the enzymes and nanozymes in immunoassays can catalyze the slow generation of gas, significantly amplifying the signal. The volume expansion or pressure increase can be monitored easily using distance readout and inexpensive hand-held devices, endowing such immunoassays with great potential in practical applications.

Although gas-generating-reaction-based immunoassays have been successfully utilized in various fields, there are still several major challenges that need to be addressed to achieve better detection performance. First, nanozymes with high catalytic activity play a critical role in gas generation for immunoassays. Despite their excellent catalytic activity with respect to gas-generating reactions, PtNPs suffer from several shortcomings, such as their high price, low stability in a working environment, and limited activity caused by surface modification. Thereby, it is necessary to exploit more effective nanozymes for gas-generation-based immunoassays, such as single-atom nanozymes and organic-molecule-assembled nanostructures. Second, most gas-generating-reaction-based immunoassays have been designed in a sandwich format with the use of a couple of antibodies. Efforts can be made to develop effective and stable artificial/synthetic receptors to replace antibodies, such as aptamers, lectins, borates, and molecular imprinting polymers. Third, more stable, safe, and versatile types of gas-generating reactions can be incorporated into immunoassays, and more precise pressure-sensing methods and devices are needed for higher detection sensitivity. Benefiting from the rapid development of smartphones, artificial intelligence, and machine learning technology, the construction of POC testing methods based on intelligent gas-generating reactions will greatly expand the application scope of immunoassays.

## Figures and Tables

**Table 3:** Overview of gas-generating-reaction-based immunoassays according to the change in solution color or temperature.

Signal Label	Target	Linear Range	Detection Limit	Ref.
PtNPs	CEA	0.2−50 ng/mL	94 pg/mL	[118]
PtNPs	CEA	0.3−60 ng/mL	0.13 ng/mL	[119]
Pt/Zn-TCPP	CEA	0.5–100 ng/mL	0.24 ng/mL	[120]
Fe_2_O_3_ particles	CRP	0.75–12 μg/mL	0.45 μg/mL	[121]
Nb_2_C MXene/Ag-Sx	HE4	10^−7^–0.1 ng/mL	3.01 × 10^−7^ ng/mL	[122]
Fe-MOF/PtNPs	*Salmonella*	1–10^7^ CFU/mL	93 CFU/mL	[123]

Abbreviation: PtNPs, platinum nanoparticles; CEA, carcinoembryonic antigen; CRP, C-reactive protein; HE4, human epididymis protein 4; TCPP, meso-tetrakis(4-carboxyphenyl)-porphyrin.

## Data Availability

Data are contained within the article.

## References

[1] Zhao Q., Lu D., Zhang G., Zhang D., Shi X. (2021). Recent improvements in enzyme-linked immunosorbent assays based on nanomaterials. Talanta.

[2] Farka Z., Juřík T., Kovář D., Trnková L., Skládal P. (2017). Nanoparticle-based immunochemical biosensors and assays: Recent advances and challenges. Chem. Rev..

[3] Liu L., Hao Y., Deng D., Xia N. (2019). Nanomaterials-based colorimetric immunoassays. Nanomaterials.

[4] Liu L., Chang Y., Lou J., Zhang S., Yi X. (2023). Overview on the development of alkaline-phosphatase-linked optical immunoassays. Molecules.

[5] Xia N., Liu G., Chen Y., Wu T., Liu L., Yang S., Li Y. (2024). Magnetically-assisted electrochemical immunoplatform for simultaneous detection of active and total prostate-specific antigen based on proteolytic reaction and sandwich affinity analysis. Talanta.

[6] Gao F., Chang Y., Xia N., Li Y., Liu L. (2024). Multifunctional self-assembled nanoparticles serving as the signal labels of fluorescent immunoassays. Microchim. J..

[7] Yang S.-M., Lv S., Zhang W., Cui Y. (2022). Microfluidic point-of-care (POC) devices in early diagnosis: A review of opportunities and challenges. Sensors.

[8] Liu L., Ma X., Chang Y., Guo H., Wang W. (2023). Biosensors with boronic acid-based materials as the recognition elements and signal labels. Biosensors.

[9] Ma X., Hao Y., Dong X., Xia N. (2023). Biosensors with metal ion–phosphate chelation interaction for molecular recognition. Molecules.

[10] Zhu L., Chang Y., Li Y., Qiao M., Liu L. (2023). Biosensors based on the binding events of nitrilotriacetic acid–metal complexes. Biosensors.

[11] Tian T., Chen G.Y., Zhang H., Yang F.Q. (2021). Personal glucose meter for alpha-glucosidase inhibitor screening based on the hydrolysis of maltose. Molecules.

[12] Abardia-Serrano C., Miranda-Castro R., de-Los-Santos-Alvarez N., Lobo-Castanon M.J. (2020). New uses for the personal glucose meter: Detection of nucleic acid biomarkers for prostate cancer screening. Sensors.

[13] Wang S., Zhou Z., Cao M., Pan Y., Zhang Y., Fang Y., Sun Q., Lei X., Le T. (2024). A comprehensive review of aptamer screening and application for lateral flow strip: Current status and future perspectives. Talanta.

[14] Hong G., Rui G., Zhang D., Lian M., Yang Y., Chen P., Yang H., Guan Z., Chen W., Wang Y. (2019). A smartphone-assisted pressure-measuring-based diagnosis system for acute myocardial infarction diagnosis. Int. J. Nanomed..

[15] He K., Xing S., Shen Y., Jin C. (2022). A flexible optical gas pressure sensor as the signal readout for point-of-care immunoassay. Analyst.

[16] Liu D., Tian T., Chen X., Lei Z., Song Y., Shi Y., Ji T., Zhu Z., Yang L., Yang C. (2018). Gas-generating reactions for point-of-care testing. Analyst.

[17] Liu X., Wang Y., Gao Y., Song Y. (2021). Gas-propelled biosensors for quantitative analysis. Analyst.

[18] Shi L., Yang C., Jin Y. (2024). Advances in gas pressure-based portable biosensing. TrAC Trends Anal. Chem..

[19] Qu K., Morioka K., Nakamura K., Yamamoto S., Hemmi A., Shoji A., Nakajima H. (2023). Development of a C-reactive protein quantification method based on flow rate measurement of an ink solution pushed out by oxygen gas generated by catalase reaction. Microchim. Acta.

[20] Huang Y., Lin C., Luo F., Qiu B., Guo L., Lin Z., Chen G. (2019). Ultrasensitive and portable assay for lead(II) Ions by electronic balance as a readout. ACS Sens..

[21] Ma X., Wang Z., He S., Chen C., Luo F., Guo L., Qiu B., Lin Z., Chen G., Hong G. (2019). Development of an immunosensor based on the exothermic reaction between H_2_O and CaO using a common thermometer as readout. ACS Sens..

[22] Wu Y., Darland D.C., Zhao J.X. (2021). Nanozymes-hitting the biosensing “target”. Sensors.

[23] Niu X., Liu B., Hu P., Zhu H., Wang M. (2022). Nanozymes with multiple activities: Prospects in analytical sensing. Biosensors.

[24] Wang K., Meng X., Yan X., Fan K. (2024). Nanozyme-based point-of-care testing: Revolutionizing environmental pollutant detection with high efficiency and low cost. Nano Today.

[25] Zhou L., Liu Y., Lu Y., Zhou P., Lu L., Lv H., Hai X. (2022). Recent advances in the immunoassays based on nanozymes. Biosensors.

[26] Shi L., Tang Q., Yang B., Li B., Yang C., Jin Y. (2023). Acid-accelerated hydrolysis of NaBH4: A gas-generation reaction for diverse gas pressure biosensing. Microchim. Acta.

[27] Yan J.M., Zhang X.B., Akita T., Haruta M., Xu Q. (2010). One-step seeding growth of magnetically recyclable Au@co core-shell nanoparticles: Highly efficient catalyst for hydrolytic dehydrogenation of ammonia borane. J. Am. Chem. Soc..

[28] Ding E., Hai J., Li T., Wu J., Chen F., Wen Y., Wang B., Lu X. (2017). Efficient hydrogen-generation CuO/Co_3_O_4_ heterojunction nanofibers for sensitive detection of cancer cells by portable pressure meter. Anal. Chem..

[29] Wang Z., Hai J., Li T., Ding E., He J., Wang B. (2018). Pressure and fluorescence dual signal readout CuO-NiO/C heterojunction nanofibers-based nanoplatform for imaging and detection of target cancer cells in blood. ACS Sustain. Chem. Eng..

[30] Tzani M.A., Gioftsidou D.K., Kallitsakis M.G., Pliatsios N.V., Kalogiouri N.P., Angaridis P.A., Lykakis I.N., Terzidis M.A. (2021). Direct and indirect chemiluminescence: Reactions, mechanisms and challenges. Mol. Cells.

[31] Barni F., Lewis S.W., Berti A., Miskelly G.M., Lago G. (2007). Forensic application of the luminol reaction as a presumptive test for latent blood detection. Talanta.

[32] Liu S., Lu S., Sun S., Hai J., Meng G., Wang B. (2021). NIR II light-response Au nanoframes: Amplification of a pressure- and temperature-sensing strategy for portable detection and photothermal therapy of cancer cells. Anal. Chem..

[33] Su L., Liu B., Su Y., Tang D. (2023). NIR II light response-based PDA/AuPt@CuS composites: Simultaneous readout of temperature and pressure sensing strategy for portable detection of pathogenic bacteria. Talanta.

[34] Hu S., Tong L., Wang J., Yi X., Liu J. (2019). NIR light-responsive hollow porous gold nanospheres for controllable pressure-based sensing and photothermal therapy of cancer cells. Anal. Chem..

[35] Alatawi F.S., Monier M., Elsayed N.H. (2018). Amino functionalization of carboxymethyl cellulose for efficient immobilization of urease. Int. J. Biol. Macromol..

[36] Talat M., Singh A.K., Srivastava O.N. (2011). Optimization of process variables by central composite design for the immobilization of urease enzyme on functionalized gold nanoparticles for various applications. Bioprocess Biosyst. Eng..

[37] Yuan Y., He Y., Pei D., Tong L., Hu S., Liu L., Yi X., Wang J. (2022). Urease-functionalized near-infrared light-responsive gold nanoflowers for rapid detection of urea by a portable pressure meter. Microchem. J..

[38] Xiao J., Zhuo D., Tang J., Chen J., Tan C., Zhang S., Li S., Zou Z. (2024). Selective and sensitive detection of cyclamate in beverages using a portable pressure meter: A specific reaction-based analytical kit. Microchem. J..

[39] Wang K.Y., Bu S.J., Ju C.J., Han Y., Ma C.Y., Liu W.S., Li Z.Y., Li C.T., Wan J.Y. (2019). Disposable syringe-based visual immunotest for pathogenic bacteria based on the catalase mimicking activity of platinum nanoparticle-concanavalin A hybrid nanoflowers. Microchim. Acta.

[40] Fu Q., Wu Z., Li J., Wu Z., Zhong H., Yang Q., Liu Q., Liu Z., Sheng L., Xu M. (2019). Quantitative assessment of disease markers using the naked eye: Point-of-care testing with gas generation-based biosensor immunochromatographic strips. J. Nanobiotechnol..

[41] Song Y., Lin B., Tian T., Xu X., Wang W., Ruan Q., Guo J., Zhu Z., Yang C. (2019). Recent progress in microfluidics-based biosensing. Anal. Chem..

[42] Liu D., Wang Y., Li X., Li M., Wu Q., Song Y., Zhu Z., Yang C. (2022). Integrated microfluidic devices for in vitro diagnostics at point of care. Aggregate.

[43] Han H., Choi S.-J. (2021). Development of an inkless, visual volumetric chip operated with a micropipette. BioChip J..

[44] Choi H.S., Jang Y.-H., Choi S.-J. (2022). Development of an Integrated Biochip System Consisting of a Magnetic Particle Washing Station and a Markerless Volumetric Biochip. BioChip J..

[45] Samadi Khezri M., Housaindokht M.R., Firouzi M. (2024). Designing and prototyping a novel biosensor based on a volumetric bar-chart chip for urea detection. Lab Chip.

[46] Yang J., Liu X., Pan Y., Yang J., He B., Fu Y., Song Y. (2019). A self-powered microfluidic chip integrated with fluorescent microscopic counting for biomarkers assay. Sens. Actuators B Chem..

[47] Bianco M., Zizzari A., Perrone E., Mangiullo D., Mazzeo M., Viola I., Arima V. (2024). Catalase detection via membrane-based pressure sensors. Molecules.

[48] Song Y., Zhang Y., Bernard P.E., Reuben J.M., Ueno N.T., Arlinghaus R.B., Zu Y., Qin L. (2012). Multiplexed volumetric bar-chart chip for point-of-care diagnostics. Nat. Commun..

[49] Cai G., Zheng L., Liao M., Li Y., Wang M., Liu N., Lin J. (2019). A microfluidic immunosensor for visual detection of foodborne bacteria using immunomagnetic separation, enzymatic catalysis and distance indication. Microchim. Acta.

[50] Li Y., Xuan J., Xia T., Han X., Song Y., Cao Z., Jiang X., Guo Y., Wang P., Qin L. (2015). Competitive volumetric bar-chart chip with real-time internal control for point-of-care diagnostics. Anal. Chem..

[51] George P. (1947). Reaction between catalase and hydrogen peroxide. Nature.

[52] Gao L., Zhuang J., Nie L., Zhang J., Zhang Y., Gu N., Wang T., Feng J., Yang D., Perrett S. (2007). Intrinsic peroxidase-like activity of ferromagnetic nanoparticles. Nat. Nanotechnol..

[53] Farka Z., Brandmeier J.C., Mickert M.J., Pastucha M., Lacina K., Skládal P., Soukka T., Gorris H.H. (2024). Nanoparticle-based bioaffinity assays: From the research laboratory to the market. Adv. Mater..

[54] Zhang M., Guo X. (2022). Gold/platinum bimetallic nanomaterials for immunoassay and immunosensing. Coordin. Chem. Rev..

[55] Xia N., Liu G., Zhang S., Shang Z., Yang Y., Li Y., Liu L. (2022). Oxidase-mimicking peptide-copper complexes and their applications in sandwich affinity biosensors. Anal. Chim. Acta.

[56] Liu X., Wang Y., Song Y. (2018). Visually multiplexed quantitation of heavy metal ions in water using volumetric bar-chart chip. Biosen. Bioelectron..

[57] Zhu Z., Guan Z., Jia S., Lei Z., Lin S., Zhang H., Ma Y., Tian Z.Q., Yang C.J. (2014). Au@Pt nanoparticle encapsulated target-responsive hydrogel with volumetric bar-chart chip readout for quantitative point-of-care testing. Angew. Chem..

[58] Song Y., Wang Y., Qin L. (2013). A multistage volumetric bar chart chip for visualized quantification of DNA. J. Am. Chem. Soc..

[59] Song Y., Wang Y., Qi W., Li Y., Xuan J., Wang P., Qin L. (2016). Integrative volumetric bar-chart chip for rapid and quantitative point-of-care detection of myocardial infarction biomarkers. Lab Chip.

[60] Li Y., Xuan J., Song Y., Wang P., Qin L. (2015). A microfluidic platform with digital readout and ultra-low detection limit for quantitative point-of-care diagnostics. Lab Chip.

[61] Wang Y., Zhu G., Qi W., Li Y., Song Y. (2016). A versatile quantitation platform based on platinum nanoparticles incorporated volumetric bar-chart chip for highly sensitive assays. Biosens. Bioelectron..

[62] Liu D., Li X., Zhou J., Liu S., Tian T., Song Y., Zhu Z., Zhou L., Ji T., Yang C. (2017). A fully integrated distance readout ELISA-Chip for point-of-care testing with sample-in-answer-out capability. Biosens. Bioelectron..

[63] Li Z., Chen H., Wang P. (2019). Lateral flow assay ruler for quantitative and rapid point-of-care testing. Analyst.

[64] Shao N., Han X., Song Y., Zhang P., Qin L. (2019). CRISPR-Cas12a coupled with platinum nnoreporter for visual quantification of SNVs on a volumetric bar-chart chip. Anal. Chem..

[65] Huang T., Yang J., Zhou W., Liu X., Pan Y., Song Y. (2019). Rapid identification of urinary tract infections based on ultrasensitive bacteria detection using volumetric bar-chart chip. Sens. Actuators B Chem..

[66] Song Y., Xia X., Wu X., Wang P., Qin L. (2014). Integration of platinum nanoparticles with a volumetric bar-chart chip for biomarker assays. Angew. Chem. Int. Ed..

[67] Li J., Bi W., Gao Y., Qin S., Yang J., Song Y., He B. (2024). Bacteria proliferation-mediated cascade amplification for visually ultrasensitive detection of extracellular vesicles. Sens. Actuators B Chem..

[68] Fu G., Zhou W., Li X. (2020). Remotely tunable microfluidic platform driven by nanomaterial-mediated on-demand photothermal pumping. Lab Chip.

[69] Zhou W., Fu G., Li X. (2021). Detector-free photothermal bar-chart microfluidic chips (PT-chips) for visual quantitative detection of biomarkers. Anal. Chem..

[70] Lee S., Kwon D., Yim C., Jeon S. (2015). Facile detection of Troponin I using dendritic platinum nanoparticles and capillary tube indicators. Anal. Chem..

[71] Wu Z., Fu Q., Yu S., Sheng L., Xu M., Yao C., Xiao W., Li X., Tang Y. (2016). Pt@AuNPs integrated quantitative capillary-based biosensors for point-of-care testing application. Biosens. Bioelectron..

[72] Li X., Dong S., Arul P., Liu H., Liu L., Wang H., Zhang Q., Gyimah E., Yakubu S., Zhang Z. (2020). A novel and facile immunosensor based on a barometer: Application for rapid analysis of *Escherichia coli* in waters. Talanta.

[73] Liu S., Lin D., Lai Y., Hou L., Lin T., Zhao S. (2022). Gas-mediated immunoassay for the carcinoembryonic antigen at atmospheric pressure with smartphone coupling with the fluorescence quenching length of perovskite capillary. Anal. Chem..

[74] Ding E., Hai J., Chen F., Wang B. (2018). Constructing 2D nanosheet-assembled MnCo_2_O_4_ nanotubes for pressure and colorimetric dual-signal readout detection of cancer cells in serum samples. ACS Appl. Nano Mater..

[75] Deng Y., Zhang C., Lv L., Wang K., Liu F., Zhou Y., Peng Z., Wang B. (2024). In situ detection of silk fibroin using a dual recognition strategy with a flexible pressure immunosensor. Anal. Methods.

[76] Bu S., Wang K., Ju C., Wang C., Li Z., Hao Z., Shen M., Wan J. (2019). Point-of-care assay to detect foodborne pathogenic bacteria using a low-cost disposable medical infusion extension line as readout and MnO_2_ nanoflowers. Food Control.

[77] Chen M., Qiu Q., Qileng A., Shen H., Liu W., Liu Y. (2023). Efficient nanozyme-triggered pressure sensor for point-of-care immunoassay: Visual sensing and time readout device. Anal. Chem..

[78] Liu L., Liu J., Huang H., Li Y., Zhao G., Dou W. (2019). A quantitative foam immunoassay for detection of *Escherichia coli* O157:H7 based on bimetallic nanocatalyst-gold platinum. Microchem. J..

[79] Liu L., Zhao G., Dou W. (2020). An unplugged and quantitative foam based immunochromatographic assay for *Escherichia coli* O157:H7 using nanozymes to catalyze hydrogen peroxide decomposition reaction. Microchem. J..

[80] Jiang H., Rao X., Li L., Liu Z. (2020). A gas pressure and colorimetric signal dual-mode strategy for sensitive detection of spermine using ssDNA-coated Au@Pt nanoparticles as the probe. Analyst.

[81] Zhang Y., Liu Q., Ma C.B., Wang Q., Yang M., Du Y. (2020). Point-of-care assay for drunken driving with Pd@Pt core-shell nanoparticles-decorated ploy(vinyl alcohol) aerogel assisted by portable pressure meter. Theranostics.

[82] Shi L., Liu W., Li B., Yang C.J., Jin Y. (2021). Multichannel paper chip-based gas pressure bioassay for simultaneous detection of multiple microRNAs. ACS Appl. Mater. Interfaces.

[83] Shi L., Lei J., Zhang B., Li B., Yang C.J., Jin Y. (2018). Ultrasensitive and facile detection of microRNA via a portable pressure meter. ACS Appl. Mater. Interfaces.

[84] Yang W., Li R., Wang Q., Wei Q., Fu C., Lin Z., Chen G. (2015). A micro-pressure sensor-based analytic platform and its application in thrombin quantification. Anal. Methods.

[85] Shi L., Tang Q., Yang B., Liu W., Li B., Yang C., Jin Y. (2022). Portable and label-free sensor array for discriminating multiple analytes via a handheld gas pressure meter. Anal. Chem..

[86] Liu D., Yu X., Li C., Wang Y., Huang C., Li M., Huang Y., Yang C. (2024). Au-Pt coating improved catalytic stability of Au@AuPt nanoparticles for pressure-based point-of-care detection of *Escherichia coli* O157:H7. ACS Appl. Mater. Interfaces.

[87] Lin B., Guan Z., Song Y., Song E., Lu Z., Liu D., An Y., Zhu Z., Zhou L., Yang C. (2018). Lateral flow assay with pressure meter readout for rapid point-of-care detection of disease-associated protein. Lab Chip.

[88] Song Y., An Y., Liu W., Hou W., Li X., Lin B., Zhu Z., Ge S., Yang H.H., Yang C. (2017). Centrifugal micropipette-tip with pressure signal readout for portable quantitative detection of myoglobin. Chem. Commun..

[89] Zhang W., Wu W., Cai C., Hu X., Li H., Bai Y., Zhang Z., Li P. (2020). A sensitive, point-of-care detection of small molecules based on a portable barometer: Aflatoxins in agricultural products. Toxins.

[90] Tao Q., Wu X., Lin Q., Zheng H., Yang W., Liu D., Yang C.J., Ji T. (2018). Portable detection of serum HER-2 in breast cancer by a pressure-based platform. Anal. Bioanal. Chem..

[91] Bu S.J., Wang K.Y., Bai H.S., Leng Y., Ju C.J., Wang C.Y., Liu W.S., Wan J.Y. (2019). Immunoassay for pathogenic bacteria using platinum nanoparticles and a hand-held hydrogen detector as transducer. Application to the detection of *Escherichia coli* O157:H7. Microchim. Acta.

[92] Zhu Z., Guan Z., Liu D., Jia S., Li J., Lei Z., Lin S., Ji T., Tian Z., Yang C.J. (2015). Translating molecular recognition into a pressure signal to enable rapid, sensitive, and portable biomedical analysis. Angew. Chem..

[93] Liu D., Liu F., Huang Y., Song Y., Zhu Z., Zhou S.F., Yang C. (2019). Catalase-linked immunosorbent pressure assay for portable quantitative analysis. Analyst.

[94] Wang Q., Li R., Shao K., Lin Y., Yang W., Guo L., Qiu B., Lin Z., Chen G. (2017). A portable immunosensor with differential pressure gauges readout for alpha fetoprotein detection. Sci. Rep..

[95] Ji T., Liu D., Liu F., Li J., Ruan Q., Song Y., Tian T., Zhu Z., Zhou L., Lin H. (2016). A pressure-based bioassay for the rapid, portable and quantitative detection of C-reactive protein. Chem. Commun..

[96] Fu Q., Wu Z., Du D., Zhu C., Lin Y., Tang Y. (2017). Versatile barometer biosensor based on Au@Pt core/shell nanoparticle probe. ACS Sens..

[97] Tang X., Wu J., Wu W., Zhang Z., Zhang W., Zhang Q., Zhang W., Chen X., Li P. (2020). Competitive-type pressure-dependent immunosensor for highly sensitive detection of diacetoxyscirpenol in wheat via monoclonal antibody. Anal. Chem..

[98] Park J. (2022). Lateral flow immunoassay reader technologies for quantitative point-of-care testing. Sensors.

[99] Xue J., Yang H., Li J., Ouyang H., Fu Z. (2023). Smartphone-based pressure signal readout device combined with bidirectional immunochromatographic test strip for dual-analyte detection. Anal. Chem..

[100] Jiang S., Zhang L., Li J., Ouyang H., Fu Z. (2021). Pressure/colorimetric dual-readout immunochromatographic test strip for point-of-care testing of aflatoxin B1. Talanta.

[101] Zeng R., Luo Z., Zhang L., Tang D. (2018). Platinum nanozyme-catalyzed gas generation for pressure-based bioassay using polyaniline nanowires-functionalized graphene oxide framework. Anal. Chem..

[102] Huang L., Zeng R., Tang D., Cao X. (2022). Bioinspired and multiscale hierarchical design of a pressure sensor with high sensitivity and wide linearity range for high-throughput biodetection. Nano Energy.

[103] Yu Z., Cai G., Tong P., Tang D. (2019). Saw-toothed microstructure-based flexible pressure sensor as the signal readout for point-of-care immunoassay. ACS Sens..

[104] Yu Z., Cai G., Liu X., Tang D. (2020). Platinum nanozyme-triggered pressure-based immunoassay using a three-dimensional polypyrrole foam-based flexible pressure sensor. ACS Appl. Mater. Interfaces.

[105] Zhu L., Lv Z., Yin Z., Li M., Tang D. (2021). Digital multimeter-based point-of-care immunoassay of prostate- specific antigen coupling with a flexible photosensitive pressure sensor. Sens. Actuators B Chem..

[106] Chen J., Xue F., Yu Z., Huang L., Tang D. (2020). A polypyrrole-polydimethylsiloxane sponge-based compressible capacitance sensor with molecular recognition for point-of-care immunoassay. Analyst.

[107] Yu Z., Tang Y., Cai G., Ren R., Tang D. (2019). Paper electrode-based flexible pressure sensor for point-of-care immunoassay with digital multimeter. Anal. Chem..

[108] Li J., Xue J., Zhang Y., He Y., Fu Z. (2022). Shape-encoded functional hydrogel pellets for multiplexed detection of pathogenic bacteria using a gas pressure sensor. ACS Sens..

[109] Li J., Liu F., Zhu Z., Liu D., Chen X., Song Y., Zhou L., Yang C. (2018). In situ Pt staining method for simple, stable, and sensitive pressure-based bioassays. ACS Appl. Mater. Interfaces.

[110] Huang D., Lin B., Song Y., Guan Z., Cheng J., Zhu Z., Yang C. (2019). Staining traditional colloidal gold test strips with Pt nanoshell enables quantitative point-of-care testing with simple and portable pressure meter readout. ACS Appl. Mater. Interfaces.

[111] Wang Q., Pang H., Dong Y., Chi Y., Fu F. (2018). Colorimetric determination of glutathione by using a nanohybrid composed of manganese dioxide and carbon dots. Microchim. Acta.

[112] Wang L., Hao L., Qi W., Huo X., Xue L., Liu Y., Zhang Q., Lin J. (2020). A sensitive *Salmonella* biosensor using platinum nanoparticle loaded manganese dioxide nanoflowers and thin-film pressure detector. Sens. Actuators B Chem..

[113] Hao Z., Lin X., Li J., Yin Y., Gao X., Wang S., Liu Y. (2021). Multifunctional nanoplatform for dual-mode sensitive detection of pathogenic bacteria and the real-time bacteria inactivation. Biosens. Bioelectron..

[114] Tian M., Wang J., Xiang W., Zheng Z., Luo Z., Jing S., Zheng Y., He S., Wei H., Yu C.-Y. (2024). A dual-mode portable platform with spatiotemporal temperature-pressure signal readouts for ultrasensitive quantitative determination of cancer cells. Chem. Eng. J..

[115] Chen Y., Wei J., Zhang S., Dai H., Lv L., Lin Y. (2022). Photothermal triggered clinical swab point-of-care testing diagnostics: Fluorescence-pressure multi-signal readout detection of cervical cancer biomarker. Chem. Eng. J..

[116] Gao L., Chen Y., Wei J., Zhang S., Yan J., Dai H. (2023). Photothermal enhanced pressure sensing based on multifunctional Nb_2_C MXene for portable detection of interleukin-6. Microchem. J..

[117] Huang L., Zeng Y., Liu X., Tang D. (2021). Pressure-based immunoassays with versatile electronic sensors for carcinoembryonic antigen detection. ACS Appl. Mater. Interfaces.

[118] Yu Z., Cai G., Liu X., Tang D. (2021). Pressure-based biosensor integrated with a flexible pressure sensor and an electrochromic device for visual detection. Anal. Chem..

[119] Huang L., Zeng R., Xu J., Tang D. (2022). Point-of-care immunoassay based on a multipixel dual-channel pressure sensor array with visual sensing capability of full-color switching and reliable electrical signals. Anal. Chem..

[120] Wu D., Tang J., Yu Z., Gao Y., Zeng Y., Tang D., Liu X. (2024). Pt/Zn-TCPP nanozyme-based flexible immunoassay for dual-mode pressure-temperature monitoring of low-abundance proteins. Anal. Chem..

[121] Song E., Tao Y., Shen H., Yang C., Tian T., Yang L., Zhu Z. (2022). A polypyrrole-mediated photothermal biosensor with a temperature and pressure dual readout for the detection of protein biomarkers. Analyst.

[122] Chen Y., Huang Y., Chen S., Gao L., Zhang S., Dai H., Zeng B. (2023). A pressure-colorimetric multimode system with photothermal activated multiple rolling signal amplification for ovarian cancer biomarker detection. Talanta.

[123] Guo R., Xue L., Cai G., Qi W., Liu Y., Lin J. (2021). Fe-MIL-88NH_2_ metal–organic framework nanocubes decorated with Pt nanoparticles for the detection of *Salmonella*. ACS Appl. Nano Mater..

[124] Zhang X., Li G., Wu D., Li X., Hu N., Chen J., Chen G., Wu Y. (2019). Recent progress in the design fabrication of metal-organic frameworks-based nanozymes and their applications to sensing and cancer therapy. Biosens. Bioelectron..

[125] Ali A., Ovais M., Zhou H., Rui Y., Chen C. (2021). Tailoring metal-organic frameworks-based nanozymes for bacterial theranostics. Biomaterials.

[126] Chen Y.Z., Wang Z.U., Wang H., Lu J., Yu S.H., Jiang H.L. (2017). Singlet oxygen-engaged selective photo-oxidation over Pt nanocrystals/porphyrinic MOF: The roles of photothermal effect and Pt electronic state. J. Am. Chem. Soc..

[127] Gao B., Liu H., Gu Z. (2016). An exothermic chip for point-of-care testing using a forehead thermometer as a readout. Lab Chip.

